# Rare finding of duodenal lymphangioma in a patient with *Helicobacter pylori* associated gastritis: A case report

**DOI:** 10.1097/MD.0000000000031455

**Published:** 2022-11-25

**Authors:** Summaiya Waheed, Naresh Kumar Seetlani, Fatima Ashfaq, Muhammad Junaid Tahir, Khabab Abbasher Hussien Mohamed Ahmed, Vineeta Kumari, Zohaib Yousaf

**Affiliations:** a Dr. Ruth K. M. Pfau, Civil Hospital, Karachi, Pakistan; b Dow Medical College, Karachi, Pakistan; c Nishtar Medical University and Hospital, Multan, Pakistan; d Lahore General Hospital, Lahore, Pakistan; e Faculty of Medicine, University of Khartoum, Khartoum, Sudan; f Dow University Hospital, Karachi, Pakistan; g Hamad Medical Corporation, Doha, Qatar.

**Keywords:** endoscopy, esophagogastroduodenoscopy, *Helicobacter pylori* gastritis, infection, lymphangioma, medicine

## Abstract

Lymphangiomas are lymphatic malformations most commonly found in the head, neck and axilla. Their incidence in gastrointestinal tract, especially duodenum is very rare. They are mostly congenital and are usually seen in children. Owing to their asymptomatic nature, these tumors are often diagnosed incidentally. Surgical resection is the definite treatment.

**Patient concerns:**

We represent a case of a 30-year-old male with duodenal lymphangioma with presentation of intermittent dyspepsia for 1 year, partially relieved by a proton pump inhibitor.

**Diagnoses:**

Upon physical examination, there was mild tenderness observed in the epigastrium. The rest of the physical examination was unremarkable. His complete blood count report was unremarkable. Upon a negative stool for *Helicobacter pylori* antigen test, the patient underwent an esophagogastroduodenoscopy which revealed *H pylori* gastritis and a duodenal lymphangioma.

**Interventions:**

Patient was put on triple therapy (clarithromycin, amoxicillin and omeprazole) for 14 days and his symptoms improved. The lymphangioma was not resected owing to small size.

**Outcomes:**

Patient was followed till 1 year and his symptoms had improved.

**Lessons:**

The case describes a correlation between *H pylori* gastritis and a duodenal lymphangioma. There is likely to be an association between the two and therefore, further studies are required to find out any relationship that may exist between the 2 conditions.

## 1. Introduction

Lymphangiomas are rare, benign lymphatic malformations that arise due to the failure of the individual lymphatic tissues to connect with the general lymphatic system.^[1,2]^ Lymphangiomas comprise thin-walled cysts primarily located in the neck, head, and axilla. The gastrointestinal tract is affected in less than 5% of the presentations.^[1,2]^ Lymphangiomas of the duodenum are even more infrequent.^[3]^ In addition to duodenum, these lymphangiomas have also been reported in other parts of the gastrointestinal tract such as esophagus, stomach, jejunum and colon.^[4]^ Although lymphangiomas can present at any age, 50% are observed at birth and 90% by 2 years of age.^[5]^ Therefore, their diagnosis in adults is rare.^[5]^ These tumors are mostly asymptomatic or present with nonspecific symptoms^[6]^ and are often diagnosed incidentally. Duodenal lymphangiomas are sessile^[7]^ and although, can be picked up on radiological examination where they appear as sharply marginated round-to-oval filling defect with smooth surface, their diagnosis is confirmed on histological basis from either biopsy, surgery or autopsy.^[8]^ The endoscopic view depicts them as soft polypoid masses with smooth and yellowish surfaces.^[8]^ They have also been described as submucosal tumors with white colored spots.^[2]^ Because they are submucosal tumors and the overlying mucosa appears normal, these tumors are often difficult to diagnose until excised and examined under microscope.^[8]^ Previous reports show, these lymphangiomas have only been diagnosed incidentally on an autopsy or diagnosed histologically following a surgery.^[8]^ Endoscopic biopsy can allow a preoperative diagnosis without any complications.^[8]^ As lymphangiomas are known to be benign with no malignant potential, these tumors can be subjected to minimal operation, and although the course of treatment depends on the surgeon, an endoscopic polypectomy may be enough to remove the tumor.^[8]^ If, however, polypectomy fails to remove the complete tumor, additional surgery should be carried out as incomplete excision can lead to recurrence.^[8]^ Lymphangiomas of lower intestinal tract should be considered for resection owing to their symptomatic nature, however, lymphangiomas of upper intestinal tract can be left unattended as they are only mildly discomforting, unless they cause a mechanical gut obstruction.^[9]^ We report a lymphangioma of the duodenum incidentally diagnosed in an adult male.

## 2. Case report

A 30-year-old male previously healthy presented in the outpatient department with intermittent dyspepsia and heartburn for 1 year. The pain was more marked in the epigastrium and increased with food intake. There was no relationship of the pain to the use of any specific food type. The patient used proton pump inhibitor omeprazole 20 mg twice daily for it, however, the pain was only partially relieved. He is nonsmoker, not alcoholic, with no self-medication user history and no alarming signs or symptoms such as anemia, weight loss, nights sweat, dysphagia or odynophagia.

The patient was vitally stable at the time of presentation. His pulse was 75/min, blood pressure was 120/80 mm Hg, respiratory rate was 15/min, and body temperature was 98°F. Physical examination revealed mild tenderness in epigastrium. The rest of the examination was unremarkable. His complete blood count was unremarkable (Table 1).

**Table 1:** Complete blood count.

Test(s)	Result(s)	Reference range(s)
HB	14.9 g/dL	13.0–16.5
RBC	5.3 × 10E6/μL	4.5–6.0
HCT	43%	40–52
MCV	81 fl	80–100
MCH	28 pg	27–34
MCHC	35 g%	30–35
WBC	7.2 × 10e3/μL	4.0–11.0
Platelets	256 × 10e3/μL	150–400
Neutrophils%	44%	40–75
Lymphocytes%	43%	20–45
Monocytes	10%	2–10
Eosinophils%	02%	01–06
Basophils%	01%	0–1

HB = hemoglobin, HCT = hematocrit, MCH = mean corpuscular hemoglobin, MCHC = mean corpuscular hemoglobin concentration, MCV = mean corpuscular volume, RBC = red blood cell, WBC = white blood cell.

The patient then underwent stool for *Helicobacter pylori* antigen 2 weeks after stopping the proton pump inhibitor, which came out to be negative. The patient was scheduled for an esophagogastroduodenoscopy as part of workup for chronic dyspepsia with incomplete response to the proton pump inhibitor.

**Figure 1.**
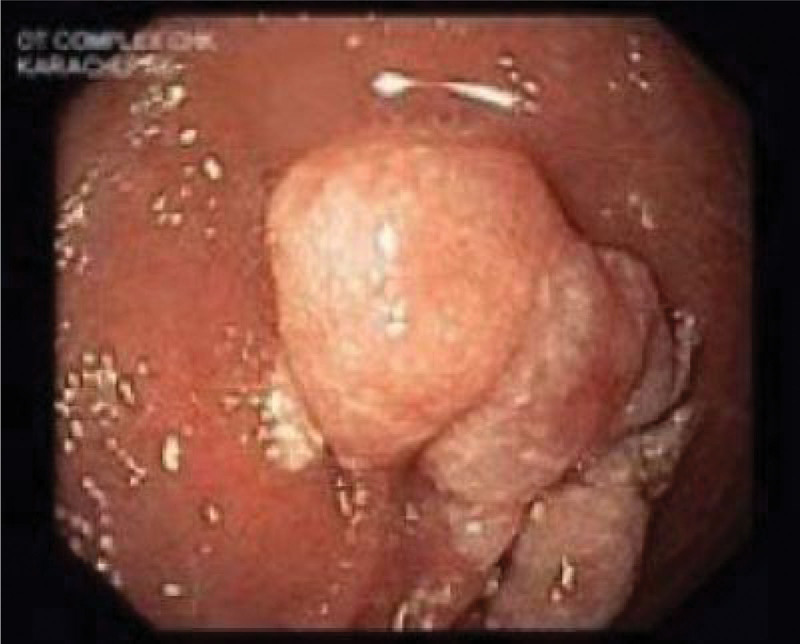
Small white polypoid mass seen at D1.

Esophagogastroduodenoscopy revealed moderate chronic active gastritis with few *H pylori* like organisms present and a small white polypoid mass was seen at D1 as shown in Figure 1. Esophagus and D2 appeared normal.

Gastric antral biopsy revealed 2 fragments of gastric antral mucosa showing moderate chronic active inflammation in the lamina propria comprising of lymphoplasmacytic cells, lymphoid aggregates, and neutrophils infiltrating into the glands. Giemsa stain revealed few *H pylori* in the lumina of the glands. No evidence of dysplasia was present.

**Figure 2.**
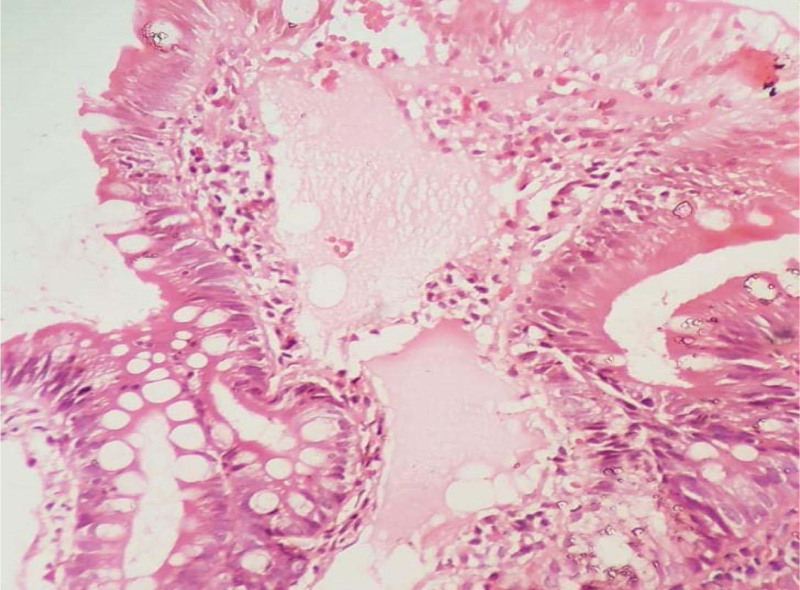
Thin-walled channels lined by flattened endothelium with eosinophilic intraluminal material seen upon 10× magnification.

The duodenal biopsy (Fig. 2) showed an intact villo-glandular architecture with an expansion of lamina propria by variably cystic vessels lined with flattened endothelial cells. Also, secretions admixed with a mixed inflammatory infiltrate were seen. Intervening stroma showed moderate inflammatory infiltrate. Underlying deeper tissues showed Brunner gland formation. There was no evidence of dysplasia, granuloma, or malignancy. All these features were suggestive of lymphangioma with moderate chronic nonspecific duodenitis.

Computerized tomography scans of the abdomen and pelvis with contrast showed distended stomach with slight diffuse increased wall thickness at the pylorus, representing collapse of pylorus. The D1 segment was partially collapsed, and hence, polypoid lesions could not be delineated. D2 and D3 segments of the duodenum appeared normal. Diffuse wall thickening involving the rectum was identified without perirectal infiltration or lymphadenopathy. The rest of the small and large bowel loops appeared normal. The rest of the scan was within normal limits. There was no evidence of lymphadenopathy seen. Abdominal and pelvic vasculature was normal, and no evidence of ascites or mesenteric fat stranding was seen. Bilateral lung bases were clear.

Confirming the diagnosis of *H pylori* gastritis, the patient was put on triple therapy consisting of clarithromycin, amoxicillin and omeprazole for 14 days. His symptoms were relieved and he was followed till 1 year. No specific treatment was done for duodenal lymphangioma.

## 3. Discussion

Lymphangiomas are rare, benign fluid-filled cyst-like lesions arising from lymphatic vessels.^[1]^ They are frequently seen in children and young adults and have an equal prevalence among both sexes.^[1]^ They can arise in any body organ except the brain, but most commonly are located in the head and neck regions (95%).^[6]^ The gastrointestinal system is rarely involved.^[3]^ Although commonly believed to arise as obstruction of lymphatic tissue that fails to unite with the systemic lymphatic system, lymphangiomas can also arise secondary to abdominal trauma, underlying inflammation, surgery, radiation exposure, or a congenital defect in the lymphatic system.^[6]^ Pathologically, they are identified as dilated lymphatics in the submucosal layer. Lymphangiomas are categorized into 3 types. Cystic is the most common form and comprises cyst-like cavities filled with protein-rich fluid. Cavernous lymphangiomas are dilated lymphatic channels of varying sizes with an intact connection with normal lymphatic vessels. Capillary lymphangiomas consist of small thin-walled lymphatic vessels beneath the skin.^[1]^

Most of these tumors are asymptomatic or have vague, nonspecific symptoms like abdominal discomfort or a palpable mass, nausea, vomiting, and dyspepsia,^[6]^ so diagnosing them can be challenging. Our patient presented with dyspepsia possibly secondary to *H pylori* infection and responded to triple therapy. However, there may be an association between lymphangiomas and dyspepsia.

Out of 567 patients who had an endoscopic biopsy performed, only 2 were reported to have benign duodenal lesions (0.35%).^[2]^ Gangl et al^[9]^ reported one lymphangioma in 2100 esophagogastroduodenoscopies, 900 colonoscopies, and 5000 rectoscopies. The second part of the duodenum is a common site of occurrence and was noticed in 63.6% of duodenal involvement.^[4]^

Although lymphangiomas of the duodenum can present with symptoms of weight loss, anorexia, anemia, bleeding, epigastric pain, they are primarily an incidental finding in asymptomatic cases because of their relatively smaller size (<1 cm).^[4]^ Duodenal lymphangiomas are submucosal tumors, often appearing as white-colored spots on the surface.^[2]^ It is believed that the accumulated chyle in the dilated lymphatics depicts itself as white spots macroscopically.^[2]^

Because of its benign nature, lymphangioma seldom requires treatment unless complicated with infection, hemorrhage, or fistula formation.^[2]^ Since our case had no such complication, no intervention was done for the lymphangioma.

Patients with dyspepsia can have an underlying lymphangioma. The association between *H pylori* and lymphangioma of the duodenum should be further investigated. Endoscopy plays a significant role in diagnosing such tumors.

## Author contributions

**Conceptualization:** Naresh Kumar Seetlani, Vineeta Kumari, Summaiya Waheed.

**Data curation:** Naresh Kumar Seetlani, Vineeta Kumari.

**Formal analysis:** Naresh Kumar Seetlani.

**Investigations:** Naresh Kumar Seetlani, Vineeta Kumari.

**Methodology:** Summaiya Waheed, Zohaib Yousaf.

**Validation:** Abdul Baqi, Summaiya Waheed, Fatima Ashfaq, Muhammad Junaid Tahir, Khabab Abbasher Hussien Mohamed Ahmed, Zohaib Yousaf.

**Writing – original draft:** Summaiya Waheed, Muhammad Junaid Tahir, Zohaib Yousaf.

**Writing – review and editing:** Zohaib Yousaf, Fatima Ashfaq, Khabab Abbasher Hussien Mohamed Ahmed, Muhammad Junaid Tahir.
